# Studying Cargo Transport Using RudLOV

**DOI:** 10.21769/BioProtoc.5468

**Published:** 2025-10-20

**Authors:** Tatsuya Tago, Takunori Satoh, Akiko K. Satoh

**Affiliations:** Graduate School of Integrated Sciences for Life, Hiroshima University, 1-7-1 Kagamiyama, Higashi-Hiroshima, Hiroshima, Japan

**Keywords:** Live imaging, Intracellular cargo transport, ER–Golgi cargo transport, Optogenetics, Confocal microscopy, RudLOV

## Abstract

Most membrane and secreted proteins are transported from the endoplasmic reticulum (ER) to the Golgi apparatus and subsequently directed to their final destinations in the cell. However, the mechanisms underlying transport and cargo sorting remain unclear. Recent advancements in optical microscopy, combined with synchronized cargo protein release methods, have enabled the direct observation of cargo protein transport. We developed a new optically synchronized cargo release method called retention using the dark state of LOV2 (RudLOV). This innovative technique offers three exceptional control capabilities: spatial, temporal, and quantitative control of cargo release. RudLOV uses illumination to trigger transport and detect cargo. Consequently, the selection of an appropriate laser and filter set for controlling the illumination and/or detection is crucial. The protocol presented here provides step-by-step guidelines for obtaining high-resolution live imaging data using RudLOV, thereby enabling researchers to investigate intracellular cargo transport with unprecedented precision and control.

Key features

• RudLOV is a new optically synchronized intracellular cargo transport method.

• RudLOV enables precise spatial, temporal, and quantitative control of cargo release.

• RudLOV allows cargo to be released using a 445 or 488 nm laser with less damage than UV.

• RudLOV allows you to hook and release cargo without the use of exogenous chemicals.

## Graphical overview


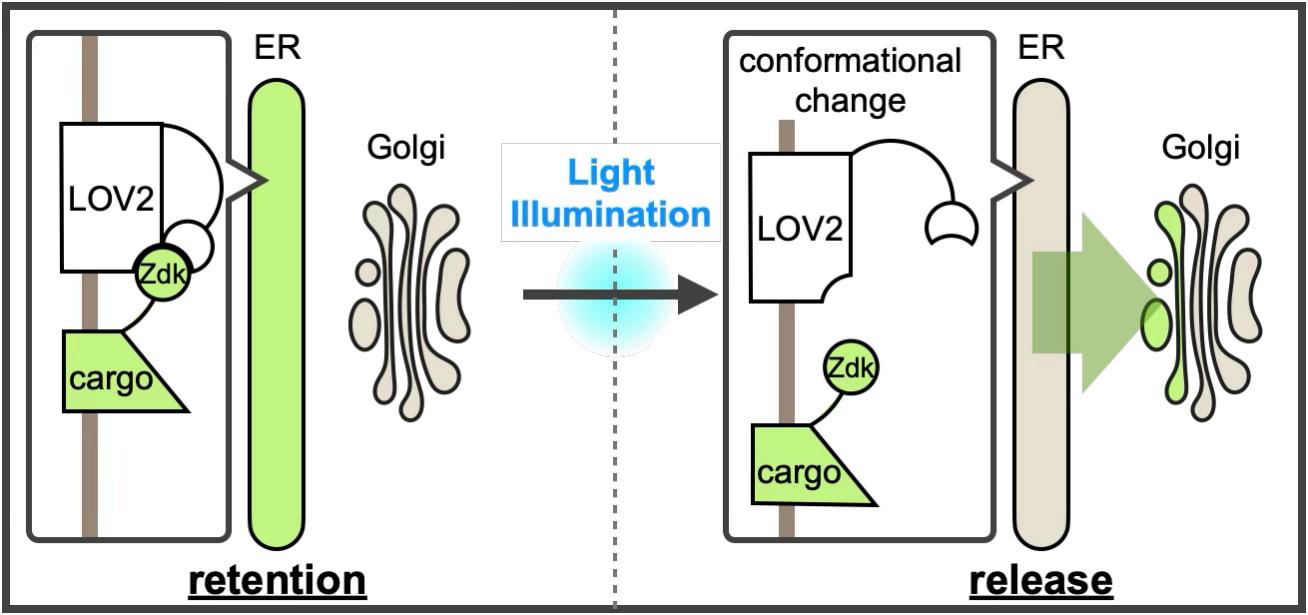


**A new optically synchronized intracellular cargo transport method, RudLOV.** In the dark, LOV2 binds to the Zdk1-fused cargo in the endoplasmic reticulum (ER). Light illumination triggers a conformational change in LOV2 that releases the cargo from the ER. RudLOV provides precise spatial and temporal control of cargo release. RudLOV functions independently of external chemicals.

## Background

Approximately 30% of the cellular proteins are synthesized in the endoplasmic reticulum (ER) and transported to the *cis*-side of the Golgi apparatus. These proteins, referred to as cargoes, traverse the Golgi apparatus and arrive at the *trans*-Golgi network (TGN), a cargo-sorting station located on the *trans*-side of the Golgi apparatus [1,2]. In the TGN, the cargo is sorted and transported to its destination or secretion. However, the mechanisms underlying the intracellular cargo transport and sorting remain unclear. Recent advances in optical microscopy, combined with methods for the synchronized release of cargo proteins fused to fluorescent proteins or tags, have enabled the real-time imaging of their journey from sequestered organelles to their destinations [3,4]. Direct observation of cargo protein transport and sorting is crucial for elucidating the mechanisms underlying these processes.

Several methods have been developed to synchronize cargo trafficking. In mammalian cells, the retention using the selective hooks (RUSH) system developed by Boncompain et al. [5] is widely used. The RUSH system is based on two key components: cargo fused to a streptavidin-binding peptide (SBP) and streptavidin fused to an organelle retention signal as a hook. The interactions between these proteins retain cargo within the organelle. The addition of biotin triggers the synchronized release of cargo proteins because it has a higher affinity for streptavidin than that of SBP. Although the RUSH system is easy to use and useful for cells that are accessible to externally applied biotin, the administration of biotin is not feasible for some cells within organisms. Furthermore, the time from biotin administration to the initiation of cargo release varies between cells. Optical cargo release methods have also been reported. In one approach, multiple UVR8-fused cargo proteins are sequestered in the ER, and a brief pulse of UV light (~310 nm) triggers cargo exit from the ER [6]. The zapalog-mediated ER trap (zapERtrap) uses UV light (405 nm) to trigger the breakdown of zapalog, which is the ER trap mechanism, allowing cargo release from the ER [7]. Both systems enable the precise control of illumination at the single-cell or subcellular level using the same microscope employed for subsequent live imaging, providing exceptional spatial and temporal accuracy for cargo release. Although these optical cargo-releasing methods are advantageous, UV light can potentially damage cells, and zapERtrap requires a continuous supply of relatively expensive zapalogs.

We recently developed a new optically synchronized cargo release method called retention using the dark state of LOV2 (RudLOV) [8], which is based on LOVTRAP (LOV2 Trap and Release of Protein) [9]. LOVTRAP is an optogenetic system consisting of LOV2 and Zdk1 tags, capable of repeatedly and reversibly controlling protein binding with precise kinetics. LOV2 is the photosensor domain of *Avena sativa* phototropin with an absorption maximum of 450 nm that utilizes the endogenous cofactor flavin mononucleotide (FMN) as a chromophore. Zdk1, an engineered aptamer that selectively binds to the dark state of LOV2, was developed by mRNA display screening of a library based on the Z-domain of protein A. LOVTRAP uses LOV2 as a trap for Zdk1-tagged proteins, which can be reversibly released by blue light illumination. LOV2 in the light state is spontaneously restored to the dark state, with a half-life of approximately 9 s in the case of wild-type *Avena sativa* LOV2.

In RudLOV, the cargo is fused to Zdk1, which is captured in the dark by LOV2 fused to ER-localized Sec61β. Mild blue light illumination (445 or 488 nm) induces a conformational change in LOV2 (-10 ms [10]), releasing Zdk1-fused cargo, which then synchronously exits the ER. The release of cargo from the ER occurs simultaneously in all illuminated cells (Figure 1F, G in Tago et al. [8]). The LOV2 conformation spontaneously reverts to the dark form within 55–81 s and recaptures the cargo with Zdk1 [11]. This mechanism allows control of the amount of cargo released by adjusting the strength and duration of illumination. We successfully demonstrated the laser power- and duration-dependent release of cargo, as well as cargo release in a single cell and even in a single Golgi stack (Figure 2A, B in Tago et al. [8]).

Live imaging requires the detection of fluorescent proteins fused to the cargo. Because both the transport trigger and cargo detection use illumination, careful selection of laser and filter sets to control illumination and/or detection is crucial for the successful implementation of RudLOV. Here, we present a step-by-step protocol for using RudLOV.

## Materials and reagents

The materials, reagents, and plasmids used have been previously described [8].

**Biological materials**

1. HeLa cells (H. sapiens) (ATCC_CCL-2)

**Plasmids**

**Figure 1.**
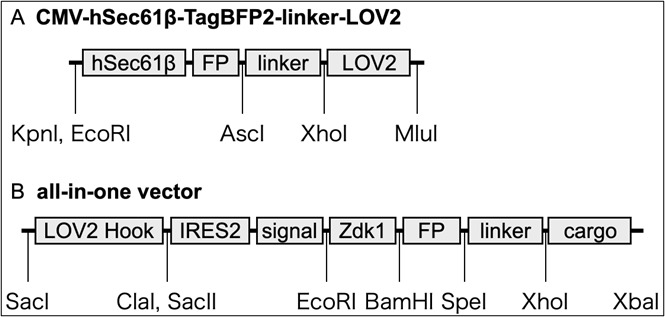
Model of RudLOV plasmids. (A) CMV-hSec61β-TagBFP2-linker-LOV2. (B) All-in-one vector. These plasmids show the major domains and unique cut sites. Abbreviations: FP, fluorescent protein (NeonGreen, Clover, Scarlet, and Halotag7); IRES2, internal ribosome entry site; signal, signal peptide (SP) and ER export signal (IPVNTT); cargo, cargo protein of interest. For more information, see Addgene.

The following plasmids were deposited at Addgene (Table 1):

1. CMV-hSec61β-TagBFP2-linker-LOV2, a plasmid vector expressing ER-hook (used only for hook/cargo-co-expression) (Figure 1a)

2. Cargo-expressing vectors such as CMV-SP-IPVNTT-Zdk1-GPI (used only for hook/cargo co-expression)

3. pEB-puro-hSec61β-TagBFP2-linker-LOV2, an EBV-based episomal vector for easy establishment of stable transformants via puromycin selection (used when establishing stable hook-expressing lines)

4. CT7-cSec61β-TagBFP2-linker-LOV2-IRES2-SP-IPVNTT-Zdk1-FluorescentProtein-cargo (all-in-one vector) (Figure 1b).

**Table 1.** List of plasmids and Addgene ID#

Addgene ID#	Plasmids	Addgene ID#	Plasmids
246600	CT7-LOVHook-Zdk-HT-GPI	246601	CT7-LOVHook-Zdk-NG-GPI
246602	CT7-LOVHook-Zdk-Sca-GPI	246603	CT7-LOVHook-Zdk-HT-VSVG
246604	CT7-LOVHook-Zdk-NG-VSVG	246605	CT7-LOVHook-Zdk-Sca-VSVG
246606	CMV-hSec61b-tBFP-LL-LOV2	246607	CMV-Zdk-HT-GPI
246608	CMV-Zdk-NG-GPI	246609	CMV-Zdk-Sca-GPI
246610	CMV-Zdk-HT-VSVG	246611	CMV-Zdk-NG-VSVG
246612	CMV-Zdk-Sca-VSVG	246613	CMV-TNFa-Zdk-HT
246614	CMV-TNFa-Zdk-NG	246615	CMV-TNFa-Zdk-Sca

The plasmid vectors for RudLOV were based on those of the RUSH system, with the Str-KDEL hook and SBP replaced with Sec61b-LOV2 and Zdk1, respectively. Similar to the RUSH system, there are separate plasmid vectors expressing hook or cargo, as well as all-in-one vectors expressing hook and cargo (see General notes 1–5).

**Reagents**

1. D-MEM (high glucose) (FUJIFILM Wako Chemicals, catalog number: 045-30285)

2. Penicillin-streptomycin-L-glutamine solution (×100) (FUJIFILM Wako Chemicals, catalog number: 161-23201)

3. 100 mmol/L sodium pyruvate (FUJIFILM Wako Chemicals, catalog number: 190-14881)

4. 1 mol/L HEPES buffer (Nacalai Tesque, catalog number: 17557-94)

5. Fetal bovine serum (Nichirei, catalog number: 174012)

6. D-PBS(-) (FUJIFILM Wako Chemicals, catalog number: 043-29791)

7. 0.25% Trypsin/EDTA (FUJIFILM Wako Chemicals, catalog number: 201-18841)

8. Nocodazole (Cayman Chemicals, catalog number: 13857)

9. Cycloheximide (Cayman Chemicals, catalog number: 14126)

10. Biotin (FUJIFILM Wako Chemicals, catalog number: 029-08713)

11. Biliverdine (TRC, catalog number: B386400)

12. JetPrime (Polyplus, catalog number: 101000015)

13. JetOptimus (Polyplus, catalog number: 101000006)

14. G418 sulfate (FUJIFILM Wako Chemicals, catalog number: 078-05961)

15. Puromycin dihydrochloride (FUJIFILM Wako Chemicals, catalog number: 160-23151)

**Solutions**

1. Cell culture medium (see Recipes)

**Recipes**

**1. Cell culture medium**

**Table:** 

Reagents	Final concentration	Volume
D-MEM (high glucose)	91%	500 mL
Fetal bovine serum	5%	25 mL
Penicillin-streptomycin-L-glutamine solution (×100)	1%	5 mL
100 mmol/L sodium pyruvate	1%	5 mL
1 mol/L HEPES buffer	2%	10 mL
Total	100%	545 mL

## Equipment

1. Inverted confocal laser scanning microscope with appropriate configuration. It is better to utilize a 514 nm laser in addition to 488 nm because 488 nm considerably activates LOV2 (see General note 9). A laser of 445 nm or similar wavelength is recommended for LOV2 conversion, although a white LED can also be used (see General note 7). We successfully performed RudLOV using the following instruments:

a. Confocal laser scanning microscope with 405/445/514/561/640 nm laser (Evident, model: FV3000). For observations without DM-switching, we strongly recommend customization with a multi-band dichroic beam splitter compatible with 445 and 514 nm lasers, such as the 405/445/514/561/640 pentaband beam splitter (Chroma 89903bs)

b. Confocal laser scanning microscope with 485–790 nm white laser + 445 nm laser (Leica, model: STELLARIS 5). This instrument has flexibility in both the excitation and detection wavelengths

2. Stage-top incubators (STXF-WSKMX-SET and STXG-IX3WX-SET)

3. Standard cell culture equipment

4. Glass bottom dish or chamber slides with coverslip bottom, such as µ-slide 8 well (ibidi, catalog number: 80826)

5. Sharp long-pass filter to block light shorter than 520 nm from transmitted illuminator, such as SC 52, 7.5 × 7.5 cm (FUJIFILM)

## Software and datasets

1. Olympus cellSens (https://evidentscientific.com/ja/products/software/cellsens)

2. Olympus FV31S-SW FLUOVIEW (https://evidentscientific.com/en/downloads)

3. Leica LAS-X (https://www.leica-microsystems.com/products/microscope-software/p/leica-las-x-ls/?country=US)

4. ImageJ/Fiji [12]

5. Volocity 6.5.1 (https://www.volocity4d.com/)

## Procedure

**A. Seed and transfect cells**

1. Plate HeLa cells in 12-well plates in 1.5 mL/well of cell culture medium (Recipe 1).

2. Wash HeLa cells 2–3 times with D-PBS(-).

3. Administer 0.25% trypsin/EDTA to HeLa cells and incubate for 1 min.

4. Suspend HeLa cells with the cell culture medium containing antibiotics and plate into the other well.

5. Culture HeLa cells in the plate for 1–2 days until they reach 80% confluence.

6. One day before transfection, seed cells at approximately 20% confluence on a glass-bottom dish or chamber slide.

7. Incubate the cells overnight at 37 °C with 5% CO_2_.

8. Set up the transfection reaction as follows:

a. 100 μL of jetOPTIMUS buffer.

b. 600 ng of plasmid (e.g., 300 ng of RudLOV construct and 300 ng of other plasmids, e.g., Golgi markers).

c. 0.9–1.2 μL of jetOPTIMUS reagent (follow manufacturer’s recommendations when other transfection reagents are used).

9. Vortex the transfection reaction and incubate at room temperature for 10 min.

10. Add 700 μL of medium to the transfection reaction, mix by pipetting thoroughly, and add to the cells.

11. Incubate the cells at 37 °C with 5% CO_2_ for 6–18 h. Avoid incubation for longer than 24 h. Subsequently, handle the cells while keeping them shaded from light (see General note 6).

12. Change the cell medium.

**B. Observation of transport within the Golgi stacks in mammalian cells using RudLOV**

1. Replace the cell medium with one containing nocodazole to disperse the Golgi stacks by inhibiting microtubule polymerization, allowing for the observation of distinct Golgi stacks.

2. Incubate the cells at 37 °C with 5% CO_2_ for at least 4 h.

3. Turn on the microscope and allow the laser and stage-top incubator to warm up for at least 30 min before observation.

4. Then, place the cells in a stage-top incubator. To prevent unintended cargo release by light from the transmitted illuminator, position a long-pass filter SC52 in the light path.

5. Setup of the acquisition experiment: Lasers with wavelengths longer than 514 nm are used to prevent accidental cargo release. For z-stack imaging of cells, it is recommended to use a 0.30 μm distance per slice, with 12–18 planes from the bottom of the cell (see General note #7).

6. Adjusting the gain and intensity settings: For example, when observing the Golgi apparatus, adjust the gain and laser intensity so that the puncta appear clear. Avoid saturation and bleaching of the fluorescence (see General note #8).

7. Start imaging.

8. Set the region of interest (ROI) and illuminate the entire cell using a 445 or 488 nm laser for 5 min or more (stimulation mode or scanning mode) (see General notes 9–12).

9. Start time-lapse imaging (see General note 13).

10. Save files as .oib/.lif.

**Figure 2.**
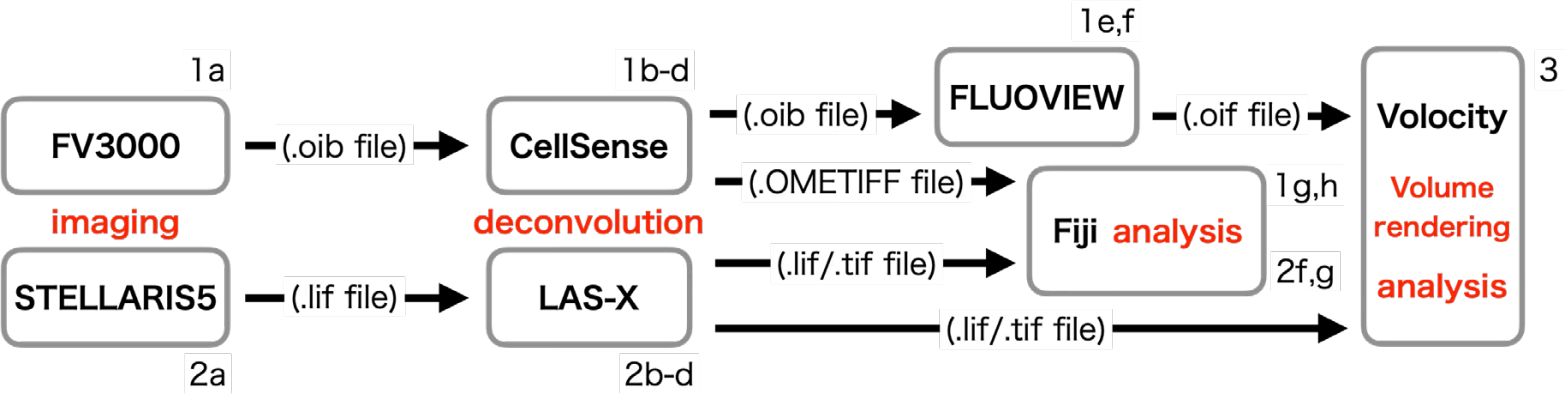
Schematic diagram of image processing. This diagram presents the flow of image processing. The alphanumeric characters near each item refer to the steps in the protocol.

**C. Processing images (Figure 2)**

1. Deconvolution and conversion of .oib files

a. Generate .oib files by imaging using FV3000.

b. Open .oib files in cellSens software.

c. Process via deconvolution using the maximum likelihood method.

d. Save the deconvoluted .oib files and export them as OME.Tiff files.

e. Open the deconvoluted .oib files in FLUOVIEW software.

f. Export as .oif files for analysis in Volocity.

g. Open these OME.Tiff files in Fiji using Bio-Formats Importer (see General note 14).

h. Save images as .Tiff files.

2. Deconvolution and conversion of .lif files.

a. Generate .lif files by imaging using STELLARIS.

b. Open .lif files in LAS-X software.

c. Process via deconvolution using Lightning Process.

d. Save processed .lif files.

f. Open these .lif files in Fiji.

g. Save images as .Tiff files.

3. Volume rendering images in Volocity

a. Initiate Volocity.

b. Make a new library.

c. Import .oif/.lif files, or create a new image sequence and drop .tif files in a library.

d. Change the color and contrast, etc.

e. Make movies/images.

f. Export movies/images as OME.Tiff/Avi.

## General notes and troubleshooting

**General notes**

**Figure 3.**
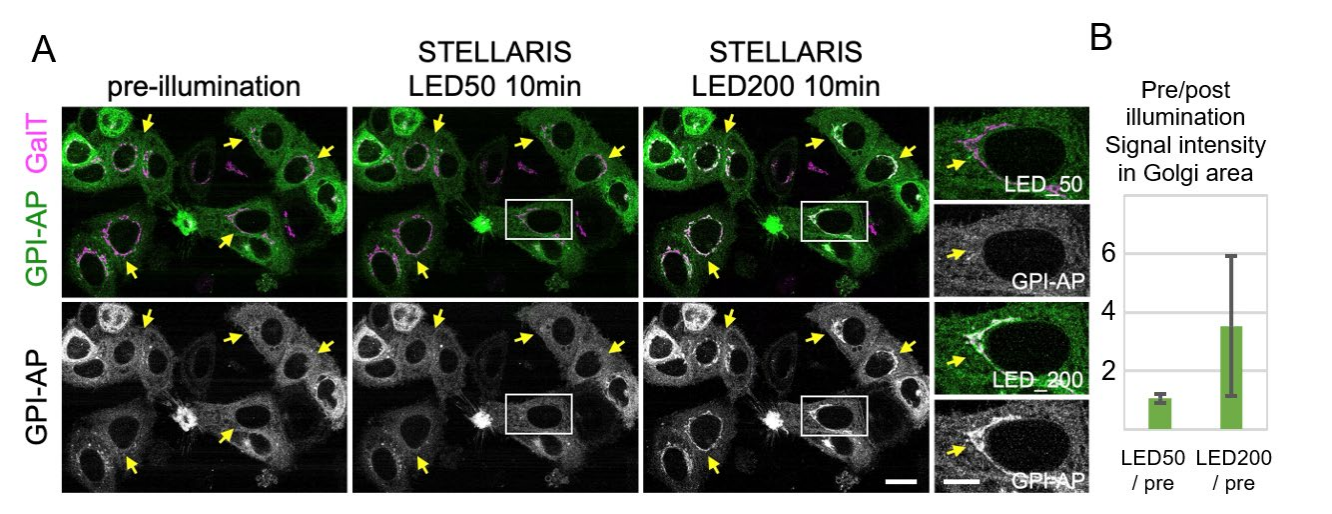
Cargo release with white LED light on STELLARIS. (A) Localization of Zdk::NeonGreen::GPI (GPI-AP: green) before and after 10 min of illumination with either LED50 light (1.7 × 10^3^ lux) or LED200 light (4.4 × 10^5^ lux). Magnified images of the insets are presented in the right panels. Arrows indicate the Golgi apparatus. HeLa cells that stably express the trans-Golgi marker GalT::iRFP713 (GalT: magenta) and the RudLOV hook construct Sec61b::LOV2 were transfected with the RudLOV cargo GPI-AP. GPI-AP was accumulated in the Golgi (GalT) in cells illuminated with LED200 light, but not in those illuminated with LED50 light. This indicates that GPI-AP exits the endoplasmic reticulum (ER) in cells illuminated with LED200 light, but not in those illuminated with LED50 light. Scale bars: 20 μm and 10 μm (insets). (B) Plots indicate the relative amounts of released cargo after 10 min of illumination with LED50 or LED200 light. Peak cargo fluorescence intensities on Golgi stacks are presented as the amount of released cargo. The signal intensity of Zdk1::Neon Green::GPI-AP on the Golgi apparatus (as defined by GalT) was measured using Fiji. Fifty-six cells were counted for each condition. Error bars are presented as mean ± SD. The values for each condition are 1.07 ± 0.16 (LED50/pre) and 3.53 ± 2.37 (LED200/pre). The result of the paired t-test is p = 2.64 × 10^-10^.

1. Localization tags should be fused to the N-terminus of LOV2 to target the ER lumen. This placement preserves the native Jα domain at the C-terminus, which is essential for the selective binding of Zdk1 to LOV2.

2. Co-transfection of separate hook and cargo vectors is possible; however, experiments can be challenging due to the varying expression levels of the hook and cargo between cells. The generation of a stable cell line expressing the RudLOV hook is highly recommended, and cells with high expression levels should be selected [8]. Alternatively, the use of all-in-one vectors that express both hooks and cargo in an appropriate balance is recommended.

3. To establish a stable hook-expressing line, transfect the cell line of interest with the EBV-based episomal vector pEB-puro-hSec61β-TagBFP2-LOV2. To establish a stable transformant, select transfected cells using 2 μg/mL of puromycin. We recommend cloning and establishing cell lines with strong hook expression.

4. Note that certain cargo types may not be released from the ER upon illumination, even if they are released by biotin administration in the RUSH system. GPI-AP, VSVG, and TNFα are retained in the ER under dark conditions and released from the ER upon illumination. However, M6PR is retained in the ER under dark conditions but is not released from the ER upon illumination, despite good release by biotin administration in the RUSH system. To date, the reason for M6PR retention in the ER after illumination is unknown.

5. ER export signals are included in the new all-in-one vector constructs, but not in the original single cargo vectors [8].

6. After transfection with the RudLOV construct, cells should be maintained in the dark. However, dim white or red light is acceptable for brief cell handling periods. We handled cells in the dark with the room light off, and they were wrapped with aluminum foil during culture.

7. As the 488 nm laser activates LOV2, wavelengths of 514 nm or longer should be used to detect cargo and organelle markers. StayGold and EGFP are not efficiently excited at these wavelengths. Slightly yellow-shifted FPs, such as mClover3 and mNeonGreen, are more suitable as green fluorescent markers.

8. For time-lapse imaging, scan each cell every 2–5 min. Therefore, the scan time is required to be within 1–2 min per cell. The laser power and gain should be set for each cell to acquire an image that is smooth and unsaturated, depending on the expression of the markers. For light illumination, set the ROI for multiple cells at a low magnification to illuminate multiple cells simultaneously.

9. Strong (but not weak) illumination with LED white light induces cargo release, enabling wide-area activation. An example of white LED illumination is shown in Figure 3.

10. LOV2 can be activated by a 405–488 nm laser; however, we recommend using longer-wavelength lasers (445 or 488 nm) because illumination with short wavelengths (405 nm) can damage the cells. Weaker laser illumination is also recommended to prevent fluorescent protein bleaching. For example, use a laser power of 0.5% during 445 nm laser illumination at 1× magnification.

11. To reduce background fluorescence and improve signal clarity from the released cargo, excess cargo in the ER can be optionally bleached out as follows: Before LOV2 stimulation, set up and save a large ROI complementary to the Golgi of interest. Stimulate LOV2 by illuminating the entire scan field with a 445 nm laser for 2–5 min to allow cargo accumulation in the Golgi. Bleach the excess cargo by illuminating the saved ROI with the laser to be used for cargo observation (typically 514 or 561 nm) with higher power.

12. Local cargo release can be induced when light illumination is applied to a limited area by setting the ROI. By limiting the illumination area to the peripheral region of the cell, it is possible to conduct research focused on local transport from the peripheral ER exit site (ERES) to the Golgi apparatus.

13. Our typical illumination and detection parameters are as follows:

a. For three-color detection, we used an FV3000 with a 445/514/561/640 nm dichroic mirror. A 445 nm laser is used for LOV2 activation. Laser wavelengths of 514, 561, and 640 nm are used to activate Clover/Neon Green, Scarlet/Ruby/mCherry, and iRFP, respectively.

b. For four-color detection, we used STELLARIS5 with a 480–790 nm white laser and a 445 nm laser. A 445 nm laser is used for LOV2 activation. Approximately 514, 560, 650, and 715 nm lasers are used to activate Clover/NeonGreen, Scarlet/Ruby/mCherry, SaraFluor 650T-Halo, and iRFP713, respectively. An example of four-color detection is shown in Figure 4.

14. When images are exported in .tiff format from the LAS-X/FLUOVIEW software, scale information is lost. Therefore, it is recommended to work with lif/OMETIFF/oir/oif files.

**Figure 4.**
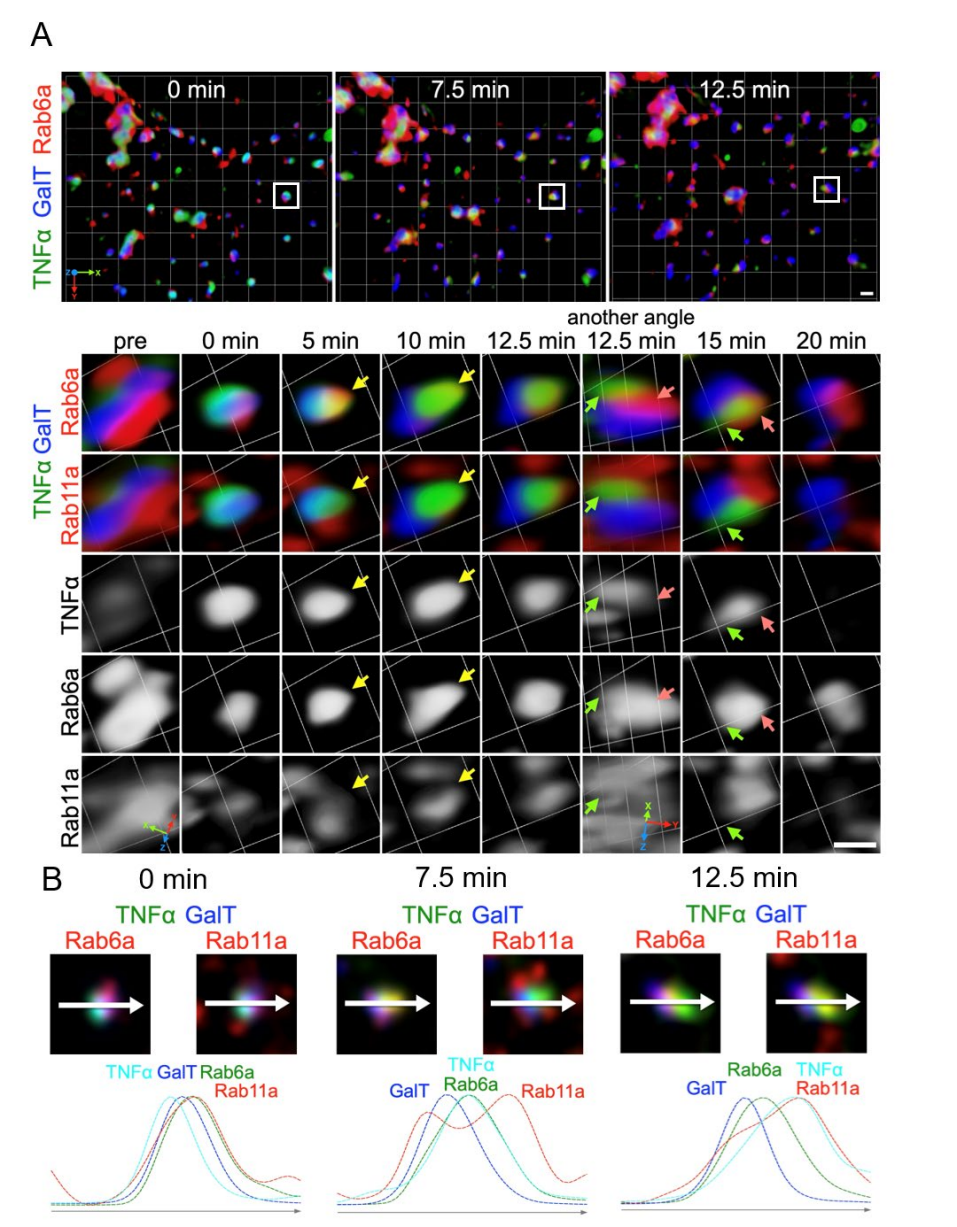
Live imaging of TNFα transport using STELLARIS in four colors. (A) Zdk1::Halo::TNFα localization labeled with SARA-Fluor 650T (TNFα) before and at 0, 5, 10, 12.5, 15, and 20 min after 10 min of 445-nm illumination. STELLARIS 5 was used for illumination with 0.2% laser power and 3× magnification. SARA-Fluor 650T was activated by a 650-nm laser, which allows visualization of TNFα localization. NeonGreen::Rab11a (a recycling endosome marker), Scarlet::Rab6a (an early TGN marker), and GalT::iRFP713 (a trans-Golgi marker) were co-expressed and activated separately using 514-, 560-, and 715-nm lasers, respectively. A 3D time-lapse volume is shown as a volumetric time series rendered using Volocity software. The upper panel presents a wide area of a cell, while the lower panel indicates a representative Golgi/endoplasmic reticulum (ER) unit. The insets in the upper panel indicate the Golgi/RE unit visualized in the lower panel. TNFα was retained in the ER before light illumination (left panels: pre). Following illumination, TNFα translocated within the Golgi stack (GalT) and subsequently reached the early TGN (Rab6a) (5–10 min, yellow arrows). Eventually, TNFα (green arrows) exited from the early TGN (red arrows) but did not enter the RE (Rab11a) (12.5–15 min). (B) Two types of tri-color images created from a single four-color image at the indicated time points indicate the relative localization of TNFα against Golgi, TGN, and RE markers. The plots below indicate the signal intensities measured along the arrow (representing 1.5 μm) on the Golgi/RE unit. Scale bars: 1 μm.

## Validation of protocol

The protocol was validated in the following research article:

- Tago et al. [8]. RudLOV is an optically synchronized cargo transport method revealing unexpected effects of dynasore. *EMBO Rep.*
